# Participation of the Cerebellum in Auditory Processing

**DOI:** 10.1016/S1808-8694(15)31076-4

**Published:** 2015-10-22

**Authors:** Patrícia Maria Sens, Clemente Isnard Ribeiro de Almeida

**Affiliations:** 1 Master's degree, the Santa Casa Medical Science College, Sao Paulo; assistant physician in the ENT clinic of the Municipal Civil Servant's Hospital (Hospital do Servidor Público Municipal).; 2 Physician. Doctorate degree, USP. Full professor in the FMJ, professor in the graduation program at the FCMSCSP. This paper is based on the author's master's dissertation presented at the Santa Casa Medical Science College, Sao Paulo, in January 2005. Address for correspondence: Rua Bartolomeu de Gusmao 412 apto. 12 Vila Mariana Sao Paulo 04111-021.

**Keywords:** cerebellum, cognition, auditory pathway, auditory perception, sound localization

## Summary

The cerebellum, traditionally conceived as a controlling organ of motricity, it is today considered an all-important integration center for both sensitivity and coordination of the various phases of the cognitive process. **Aim:** This paper aims at gather and sort literature information on the cerebellum's role in the auditory perception. **Methods:-** We have selected animal studies of both the physiology and the anatomy of the cerebellum auditory pathway, as well as papers on humans discussing several functions of the cerebellum in auditory perception. As for the literature, it has been discussed and concluded that there is evidence that the cerebellum participates in many cognitive functions related to hearing: speech generation, auditory processing, auditory memory, abstract reasoning, timing, solution of problems, sensorial discrimination, sensorial information, language processing, and linguistic operations. **Conclusion:** It has been reported that all information about cerebellum structure, functions and auditory pathways is incomplete.

## INTRODUCTION

The cerebellum has been traditionally seen as part of the motor central nervous system responsible for starting and regulating standard movements, receiving stimuli primarily from the motor cortex, vestibular organs and propioceptive receptors. ^1^,^2^

It has been reported that the phylogenesis of the human cerebellum evolved in two ways, by increasing the population of nervous cells and dendritic processes, and increasing the connections that link the cerebellum to the pre-frontal cortex, thus suggesting that the cerebellum acts as a quick adjunct in processing information together with the cerebral cortex^1^. Other authors have also observed that this connection of the cerebellum with the pre-frontal cortex, through the retrograde transneural transport of labeled type I herpes simplex virus (HSVI) in monkeys, thus providing an anatomical substrate for the cerebellum participation in the cognitive process^2^.

Studies regarding the organization of the auditory area in the cerebellum of animals started out during the 40's, when they observed evidences that in cats the cerebellum receives inputs from auditory, tactile and visual senses, transmitting information to the cortical pathways^3^. After that, many studies followed, most of them using electrophysiological exams; though, in different animal species, trying to establish the cerebellar auditory area and its central and peripheral connections^4^, ^5^, ^6^, ^7^, ^8^, ^9^, ^10^, ^11^, ^12^, ^13^.

Once they identified in animals these hearing-related cerebellar areas and pathways, studies were initiated in humans, aiming at determining the functions of these anatomical details.

Image studies using PET Scan and MRI have been important, since they use non-invasive methods in order to provide for indirect monitoring of neural activities in humans, associated to changes in blood flow and oxygenation.

In the first half of the 20th century, they started studies that indicated the possibility of the cerebellum carrying out functions other than movement coordination, and in these regards many papers have been published in the last 30 years.

Nonetheless, within the functional borders of sensory-motor integration, the cerebellum may be considered not only an important structure in motor control, but also a crucial link in integrating sensory stimuli.

The aforementioned assumptions suggest that individuals with cerebellar disorders, besides motor impairments, also have cognition disorders - hearing impairment among them, since within it we find a number of cognitive functions such as sound location, auditory temporization, auditory memory, auditory attention, amongst others. Knowledge about these functions is important in order to aid in the diagnosis of cerebellar dysfunction-related problems of hearing perception and processing, as well as to guide treatment.

Our goal with the present study is to summarize literature information as to cerebellum participation in hearing processing.

## MATERIALS AND METHODS

We made a systematic review and critical analysis of papers surveyed in the Index Medicus, electronically accessed through MEDLINE, PUBMED, LILACS, Cochrane Library, Ovidis, Scielo, digital review of the Brazilian Journal of Otorhinolaryngology, reviewing references of selected papers and text books.

Key words were determined based on the ones used in pre-selected papers that matched the inclusion criteria. The ones listed below were the ones most sensitive for paper search:

“Cerebellum”, “Auditory Perception”, “Auditory Pathway”, “Cognition”, “Sound Localization”, “Auditory Processing”.

“Cerebelo”, “Percepção auditiva”, “Localização sonora”, “Cognição”, “Vias Auditivas”, “Processamento Auditivo”.

We used texts written in English, Portuguese and Spanish.

Quality criteria to which the papers were submitted: Prognostic user's guide and the quality assessment criteria from the Brazilian Journal of Otorhinolaryngology.

Inclusion criteria: studies that mention hearing processing or hearing perception and cerebellum.

Exclusion criteria: papers that mention cerebellum cognitive function unrelated to hearing.

## RESULTS

Following inclusion and exclusion criteria, we identified 783 citations for electronic searches, 714 excluded citations based on title and abstract. In total, we found and accessed 69 texts, together with 17 texts obtained throughout references. Of these, 56 papers were excluded based on the reading of the original paper, in which hearing did not participate on the cognitive process. Therefore we primarily excluded 13 texts with studies involving research in animals and 17 texts with studies involving research in humans.

1) Animal studies:

Most authors agreed that the cerebellar hearing area is located in lobes VI and VII of Larsell^3^, ^4^, ^5^, ^6^, ^7^,^10^, ^11^, ^12^, ^13^. Some authors added some areas to these ones, such as lobes V and VIII of Larsell, paramedian lobe, the cerebellar hemisphere and the parafloccular lobe^6^,^8^,^9^,^12^. Other authors disagreed from the majority, and described the cerebellar hearing area being located in lobes VIII, IX and X of Larsell^14^.

The cerebellum peripheric afferent hearing pathways were studied by means of evoked potential tests, in which the researchers observed a threshold reduction in cerebellum response areas with the destruction of the inner ears of cats; thus showing the pathway that goes from the cochlea to the cerebellum^3^. Other authors suggested that the peripheral afferent pathway would go from the cochlear nucleus to the cerebellum, also using evoked potential tests^7^.

Central cerebellar afferent auditory pathways were suggested as going through the pontine nucleus, inferior colliculus and auditory cortex, by means of electrophysiologic exams^7^. Snider and Stowel observed that the inferior colliculus destruction would lead to an abolishment in the response areas of the cerebellar hearing area. Now, Azizi observed that the electrical stimulation of the inferior colliculus evoked responses in the cerebellar vermis, just as the electrical stimulation of the auditory cortex was able to evoke mixed responses in the cerebellar paraflocular lobe.

Cerebellar central efferent auditory pathways were assessed by the electrical stimulation of the auditory reception area of the cat's cerebellum, where they noticed the activation of the inferior colliculus, medial geniculate body and auditory cortex^10^. The same authors also described a parallel pathway, going from the cerebellar auditory area, towards the reticular formation and cortical auditory area; however, without the participation of the medial geniculate body.

Among cerebellar peripheral efferent auditory pathways described in guinea pigs and the Rhesus monkey, the cerebellar-superior olivary complex-cochlea pathway was described and also an inhibitory pathway acting on the hair cells^11^,^12^. Gacek observed through the labeling of the cochlear nucleus in cats after Wallerian degeneration of the cerebellar auditory pathway. Another study using the method of mapping the cerebellar auditory area with the peroxidase enzyme, observed the labeling of the cochlear nucleus^6^.

Most of the authors found short and average latency potentials during the tests with evoked potentials, with variations between 6 and 30 milliseconds^3^, ^4^, ^5^,^7^, ^8^, ^9^.

2) Studies in humans:

Many cerebellar cognitive functions have been described in humans:

Timing: Studies about the cerebellar timing function have noticed alterations in the time interval pair discrimination in patients with cerebellar disorders, as well as the activation of the right cerebellar hemisphere in normal individuals submitted to functional MRI during the discrimination of pauses between tone pairs, thus suggesting that the cerebellum participates in the perpetual processing of the non-verbal stimuli; and that such organ could work as a back up mechanism, expanding its capacity to store the “hearing analyzer”, extracting temporal tips from acoustic signals15-20. This way, the cerebellum would contribute to temporal organization, maintenance and monitoring, within a complex system involving multiple components from different neural regions^15^,^16^.

Auditory attention: Studies have suggested that the cerebellum would act as an intensifier of neural responses, coordinating the selective attention direction and, as a consequence, aiding in the execution of commands generated in the cortex in order to stimulate and inhibit different sources of information^21^, ^22^, ^23^. Patients with cerebellar lesion submitted to tests, used to assess hearing attention, presented impairment in the subtle change of attention between a visual and an auditory stimulus, suggesting that the cerebellum may somehow affect the voluntary control of a specific cognitive operation, thus contributing in the fast and accurate change of attention, without the participation of the cerebellar motor control function^21^,^22^.

Auditory memory: Auditory memory has been assessed by many authors in the most varied ways, from normal individuals submitted to PET Scans during short and long duration memory tests with cerebellar activation in both tasks, to patients with cerebellar lesions, studying auditory and visual memory types by means of neurobehavioral tests - assessing response impairment in these patients during auditory memory tests^16^,^17^,^24^, ^25^, ^26^. These studies have suggested that there is a multicentric cerebral neural network that participates in this function, and that such network, of which the cerebellum is one of the participants, would integrate itself in order to carry out the tasks of memory coding, storage and recovery^16^,^17^,^27^,^28^.

Some authors have assessed the auditory memory of children with cerebellar lesion and suggested that the cerebellum-cortex integration develops itself early on, and that alterations in the auditory memory, which have been observed in these patients, would spring from the interruption of the intercommunication network between the cerebellum and cortical areas, and not from cerebellar lesions in themselves^24^,^26^.

Semantic auditory processing: Semantic auditory processing has been assessed in functional neuro-image studies, in which the cerebellum was activated together with some other neural structures, such as the left posterior temporal cortex, the left inferior frontal cortex, the prefrontal cortex and the supplementary motor area. Studies involving auditory semantic and non-semantic tasks have observed a greater cerebellar activation, especially during the auditory semantic task^27^,^29^.

Language processing and linguistic operations: Studies have suggested that linguistic deficits followed by cerebellar diseases do not imply the representation of linguistic functions at the cerebellum, but rather reflect a functional deactivation of the supratentorium language areas, because of stimuli reduction on the cerebellum-cortical pathways, especially regarding the de-synchronization process and a relevant pathological mechanism cerebellum-borne language disorders. Other authors have also mentioned the modulating role of the cerebellum, highlighting that such modulation is impaired, thus linguistic deficits caused are quantitatively and qualitatively different from the deficits produced by supratentorium structure lesions. Therefore, a batch of standard tests are often times not sensitive enough to reveal alterations that may be caused by cerebellar lesions^15^,^16^,^23^,^24^.

Verbal generation: Verbal generation has been observed in the studies that revealed the difficulty in learning verbal associations and the deterioration in verbal fluency tests in patients with cerebellar lesions. Right side cerebellar activation was observed during PET Scanning while normal individuals uttered a verb, related to a substantive presented in both auditory and visual way, suggesting cerebellar participation in verbal generation cognitive function^9^,^23^, ^24^, ^25^.

Sensorial information and discrimination: It is important to highlight the close relationship between auditory and visual sensibilities, as reported by numerous authors. This knowledge is important in order to understand the multi-sensorial communication mechanism and plan patients' rehabilitation. ^26^,^30^.

Abstract reasoning, skillful handling of ideas and problem solution: These are all functions which are part of auditory processing necessary activities, therefore, if compromised it could impair such processing^1^,^15^,^24^.

## DISCUSSION

**Figure 1:**
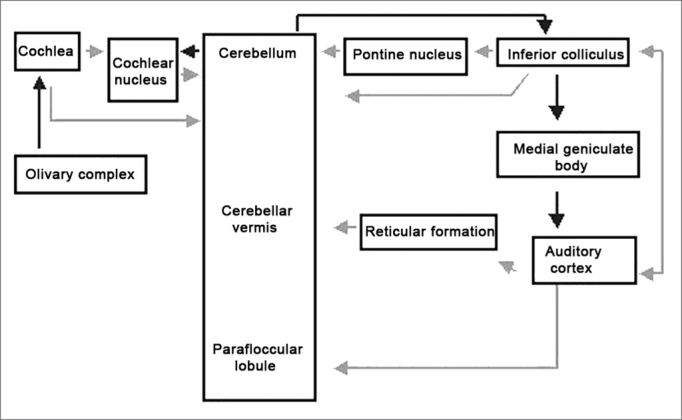
Summary of the cerebellum auditory pathways that have been anatomically identified by numerous authors

Using the numerous pieces of isolate information found in the literature, the authors assembled a scheme that allows the visualization of the different cerebellar connections with peripheral auditory organs and many central structures (Figure 1). From it we may conjure up that the cerebellum is involved in complex central activities, and most probably in peripheral functions, modulating cochlear activities - may be in an inhibitory fashion, as observed in some studies in which cerebellar lesions in animals increased cochlear microphonics amplitude and auditory nerve action potential in animals; however, with too few studies to yield a final statement. ^11^,^16^,^26^.

It is important to stress that this auditory pathway, which goes through the cerebellum, is not the only one, since there are other parallel auditory pathways that do not go through the cerebellum, such as the pathways that go from the cochlear nucleus to the inferior colliculus.

The divergence among some authors in determining the cerebellar auditory area has been observed in some studies6,8,12,14. We must bear in mind that the animals studied in such research projects vary in species, and thus they may influence results. These different findings may signify a species adaptation in order to survive

Some differences have been found in the literature in relation to the need for one or both ears to participate in the production of auditory responses in the cerebellum. Some authors have observed that the responses happened with the participation of both ears, and also that there was greater effectiveness of the binaural stimulus when compared to the monoaural one^3^,^4^. Notwithstanding, some authors have reported that longer latency neurons (longer than 11 ms) responded to the binaural stimulus, while shorter latency neurons (shorter than 6ms) responded to the monoaural stimulus^7^. Such mechanism may aid in sound location, since binaural stimulation is more effective and aids in attaining sound time and intensity differences.

Another important piece of data is that most authors who performed experimental studies in animals observed middle latency potentials during the performance of evoked potentials^3^,^5^,^7^,^8^. Such data suggests that the cerebellum acts as a collaborator in the auditory processing and in cognition, thus representing an open field for numerous research projects.

Some authors have advocated that the influence of the neo-cerebellar activity on the brain stem, thalamus, hippocampus and cerebral cortex may enhance or inhibit responses for somatosensorial, auditory and visual stimuli; notwithstanding, the importance of this sensorial modulation over behavior by the neo-cerebellum is yet unknown, as are the physiological rules that govern such function in animal behavior^15^,^30^.

Many complementary studies among themselves unveiled the cerebellar influence in the many phases of auditory processing, as mentioned above, showing that such cerebral structure, that until very recently was regarded only as a structure that coordinated movement, is extremely important for cognition and communication. Practical applications, that may develop from such knowledge are numerous and represent an open field for research, making it easy to forecast new and more adequate diagnostic methods for communication and cognition disorders, as well treatment techniques for bearers of such limiting disorders.

## CONCLUSIONS

Cerebellum auditory pathways and structures are still to be fully understood. The cerebellum acts as a modulator of numerous types of sensibility in the cognition process. The cerebellum hosts an integration of sensorial information destined to cognition, involving auditory and visual stimuli. In the literature there are evidences that the cerebellum participates in the following cognitive hearing-related functions: verbal generation, auditory attention, auditory memory, abstract reasoning, timing, problem solving, sensorial discrimination, sensorial information, language processing and linguistic operations. The cerebellum auditory activity contributes in the creation of middle latency potentials in animals and impacts the activities of the peripheral auditory efferent pathways, and this may become a research goal for practical application in humans.
